# Tryptophan 2,3-dioxygenase may be a potential prognostic biomarker and immunotherapy target in cancer: A meta-analysis and bioinformatics analysis

**DOI:** 10.3389/fonc.2022.977640

**Published:** 2022-10-03

**Authors:** Yanyan Hu, Zhongjian Liu, Hui Tang

**Affiliations:** ^1^ Department of Gastroenterology, The First People’s Hospital of Yunnan Province, The Affiliated Hospital of Kunming University of Science and Technology, Kunming, China; ^2^ Medical School, Kunming University of Science and Technology, Kunming, China

**Keywords:** tryptophan 2,3-dioxygenase, tumor, prognosis, biomarker, immune, meta-analysis

## Abstract

**Background:**

Tryptophan 2,3-dioxygenase (TDO2) is one of the emerging immune checkpoints. Meanwhile, TDO2 is also a key enzyme in the tryptophan (Trp)–kynurenine (Kyn) signaling pathway. Many studies have evaluated that TDO2 is highly expressed in various malignant tumor patients and plays a prognostic role. However, the sample size of a single prognostic study was small, and the results were still controversial.

**Methods:**

We used Stata software and referenced the Preferred Reporting Items for Systematic Reviews and Meta-analysis (PRISMA) statement to conduct a meta-analysis on TDO2 and its clinical features and prognosis. We searched the PubMed, Cochrane Library, and Web of Science databases to find publications concerning TDO2 expression in malignant tumor patients up to June 2021. We used the Newcastle–Ottawa Scale (NOS) to evaluate the bias risk of the included literature. Risk ratios (RRs) and hazard ratios (HRs) were used for clinical outcomes, specifically overall survival (OS) and progression-free survival (PFS). In addition, we used data from The Cancer Genome Atlas (TCGA) to verify our conclusions.

**Results:**

Nine studies including 667 patients with malignant tumors were identified. Our results suggested that overexpression of TDO2 was statistically correlated with poor OS and poor PFS (HR = 2.58, 95% CI = 1.52–4.40, *p* = 0.0005; HR = 2.38, 95% CI = 0.99–5.73, *p* = 0.05). In terms of clinicopathological characteristics, the overexpression level of TDO2 was statistically correlated with TNM (tumor–node–metastasis) stage (RR = 0.65, 95% CI = 0.48–0.89, *p* = 0.002) and regional lymph node metastasis (RR = 0.76, 95% CI = 0.59–0.99, *p* = 0.04). Subgroup analysis revealed the potential sources of heterogeneity. In addition, bioinformatics studies suggested that the level of TDO2 was high in malignant tumors and higher in cancer tissue than in matched paracarcinoma tissue. Gene enrichment analysis showed that TDO2 was closely related to immune response.

**Conclusion:**

Overall, TDO2 may be a biomarker for the survival and prognosis of patients with malignant tumors and a potential therapeutic target in the future.

**Systematic Review Registration:**

https://www.crd.york.ac.uk/prospero/display_record.php?RecordID=260442, identifier (CRD42021260442)

## Introduction

In recent years, immunotherapy has been suggested to be an emerging approach to treating malignant tumors (1). Immune checkpoint inhibitors (ICIs) have achieved tremendous progress in patients with malignant tumors (2, 3). Unlike traditional chemotherapeutic drugs, ICIs enhance the body’s natural tumor-killing response to protect patients (4). It is worth noting that ICIs are considered to be the fifth largest cancer treatment type, following surgery, chemotherapy, radiotherapy, and targeted therapy (5). Targeted immunotherapy is now becoming a new cancer treatment strategy, and the US Food and Drug Administration has approved many medicines, like immune checkpoint blockade agents targeting PD-1 and CTLA-4 (6, 7).

Tryptophan 2,3-dioxygenase (TDO2) plays an important role in regulating immune response and tumor immune escape (8). Previous studies have shown that tryptophan metabolism mediated by TDO2 can mediate tumor immune tolerance (9). In addition, immune escape is an important feature of malignant tumors, which involves complex mechanisms such as allowing malignant cells to evade the host immune system (10). *In vivo* experiments performed with an immunocompetent mouse model have confirmed that tumors achieve immune tolerance through the induction of TDO2 expression (11). One study confirmed that TDO2 is constitutionally expressed in many human tumor cell lines (12). These results indicate that TDO2 is a negative regulatory molecule that regulates the prognosis of malignant tumors. Additionally, inhibitors targeting TDO2 have been approved for clinical trials in the past decade (13).

Previous publications have also indicated that TDO2 is expressed in various cancers (14–16). Recent reports have shown that the level of TDO2 in the tissues of patients with malignant tumors is high (15–17). In addition, several articles have demonstrated that elevated TDO2 levels are related to poor survival (9, 18, 19). However, other studies report contrary results, showing that TDO2 levels are unrelated to tumor prognosis (11, 12, 20). Therefore, the value of TDO2 levels for survival in patients with malignant tumors is still debated.

The main purpose of this article was to explore the relationship between TDO2 and clinical features and survival outcomes in malignant tumor patients. The data from this study can enhance our understanding of TDO2 in the prognosis of human malignant cancer. In addition, it can also provide a basis for the development of TDO2-specific drugs or inhibitors.

## Methods

### Literature search

This paper was based on the Preferred Reporting Items for Systematic Reviews and Meta-analysis (PRISMA) statement (**Table S1**) (21). It has been registered with PROSPERO (ID: CRD42021260442). We searched three databases—PubMed, Web of Science, and The Cochrane Library—to find the article that studies the clinicopathological features and prognostic significance of TDO2 in malignant tumors from inception until 29 June 2021 by two independent authors (YH and ZL). We used the following MeSH and keywords to search: (“Tryptophan 2,3-Dioxygenase” OR “TDO2”) AND (“cancer” OR “carcinoma” OR “tumor” OR” neoplasm”) AND (“prognosis” OR “survival” OR “outcome” OR “prognostic”). We provided a complete electronic search strategy for the PubMed database (**Table S2**). In the process of literature inclusion, we not only searched the journals manually, but also tracked the references of related literatures. Two independent investigators (YH and ZL) performed the literature search. When there was controversy in the inclusion process, our two researchers discussed and reached a consensus decision.

### Literature inclusion criteria

The evaluation and screening of literature were carried out independently by two researchers with the same criteria, and finally cross-checked. The two researchers screened the articles according to the following inclusion requirements (1): an independent case–control or cohort study reported in the article (2); the patients were diagnosed with malignant tumors, and no other cancers were combined (3); studies evaluated the associations between the TDO2 expression level and clinicopathological features or cancer prognosis parameters, such as OS, PFS or DFS (4); the data in the paper can be used to calculate the related values of survival and prognosis, such as RR or HR and 95% confidence interval (5); the patients in the literature can be divided into a high and a low group of TDO2; and (6) the article was published in English.

### Literature exclusion criteria

The criteria for the exclusion of literature are as follows (1): the article contains duplicate data (2); studies based on animal models (3); other types of literature, such as individual case reports, reviews, conference summaries, or other non-original studies; or (4) the data in the literature are based on sequencing data or public platforms. When the selected article is controversial, the two researchers discuss it and resolve it.

### Data extraction

The two researchers extracted data from the literature according to the following criteria (1): basic information of the article such as the first author, publication year, country, sample size, cancer types, research type, cutoff value, detection methods, and the number of patients with high expression of TDO2 (2); clinical features such as age, sex, T classification, lymph node metastasis, distant metastasis, and TNM stage; and (3) HR and 95% confidence interval between TDO2 and survival indicators in patients with malignant tumor. If the HR and its 95% confidence intervals were not directly provided in the article, we contacted the authors *via* email to obtain sufficient data for calculation. If we still could not get enough information, we used the method suggested by Tierney, which was to extract data from Kaplan−Meier survival curves through Engauge digitizer software (22). Two researchers (YH and ZL) independently extracted statistical data according to consistent criteria.

### Quality assessment

The Newcastle–Ottawa Scale (NOS) was used to examine the risk of bias between studies (23). NOS is an evaluation carried out by the Cochrane Collaborative Network, a quality evaluation form included in the literature. The commonly used quality evaluation in case–control studies and cohort studies is NOS. It is a simple and convenient quality assessment tool for non-random studies selected for systematic review. The quality of the scale is represented by the score, and the full score is 9. The evaluation items mainly included three items: object selection, comparability, outcome (cohort study), or exposure (case–control). There were evaluation items under each item, and each item was expressed as 1 point at that time, of which the highest score was 2 for comparability. The final included study quality score is evaluated independently by two researchers (YH and ZL). If there is a dispute, the two researchers will discuss it to reach an agreement. The lowest score of NOS is 0 and the highest score is 9. If its average score is greater than 6, the quality of the included literature is high. If it is less than or equal to 6, then the quality of this included literature is not the best.

### Statistical analysis

We used the STATA package (version 14.0) to do this meta-analysis. For the data extracted from the literature, we used SPSS (version 22.0) software for data management and statistical calculation. The associations of TDO2 with patient OS and PFS were estimated through forest plots. We can obtain a single HR and its 95% confidence interval in each included literature. Combining these survival prognosis values, we obtained a combined HR and confidence interval. These combined values can reflect the survival and prognostic value of TDO2 and patients with malignant tumors. Furthermore, each included individual study also has an RR value and its 95% confidence interval. We used the value of RR to evaluate the association between TDO2 expression level and clinical features. Combining RR values can inform on the relationship between TDO2 expression levels and basic clinical information in patients with different malignant tumors. If the final combined HR/RR value is greater than 1, it means that the level of TDO2 is linked to the worse survival outcome; on the contrary, if the HR/RR value is less than 1, it means that the expression of TDO2 is linked to a better prognosis. If the maximum and minimum of the 95% confidence interval of HR/RR value contain 1, it means that the expression of TDO2 has no significant statistical significance on the survival outcome or clinical features of malignant tumor patients (24). The sources of heterogeneity are mainly from the following three aspects: clinical heterogeneity, diversity of methodology, and statistical sources. For the measurement of heterogeneity, we mainly used chi-square-based Cochran’s Q statistics (25). As for the choice of benefits model, it is divided into the random-effects model and the fixed-effects model. Due to clinical and methodological heterogeneity across articles, we used a random-effects model to reduce sources of heterogeneity in our statistics. When the *p*-value of the *Q* test is less than 0.05, it indicates that there is heterogeneity in the outcome of our study. At this time, we performed a subgroup analysis of meta, which usually refers to the analysis of a certain characteristic of the object, such as sex, age, or subtype of the disease, to explain the source of heterogeneity. Subgroup analysis was conducted based on cutoff value, sample size, NOS score, HR sources, study country, and year of publication. Moreover, we did a sensitivity analysis, which could test the robustness of the final outcome. Sensitivity analysis is a common method in meta-analysis, which mainly excludes each included study one by one, and then combines the outcomes to observe the impact. If the results of the merger are still statistically significant, it shows that our results are robust. If the results of the merger are not statistically significant, then our results need to be interpreted carefully. If the *p*-value of the result is less than 0.05, it is statistically significant; otherwise, it is not statistically significant. We used subjective a funnel plot and quantitative Begg test for publication bias (26). The funnel plot looks like an inverted triangle, with large samples at the top and small samples at the bottom. We can see whether there is publication bias by visual symmetry. If both sides of the graph show symmetry, it indicates that there is publication bias in the outcome of the study; otherwise, it indicates that there is publication bias in the outcome of the study. Furthermore, Begg test can use qualitative methods to determine whether there is publication bias. For the test of publication bias, if the *p*-value is <0.05, it means that there is publication bias; otherwise, it means that no publication bias was found (27).

### Metatrim

The method was proposed by Taylor and Tweedie. The basic idea is to first cut out the asymmetrical part of the funnel diagram after the initial estimation, estimate the central value of the funnel diagram with the remaining symmetrical part, and then paste the cut part and the corresponding omitted part along both sides of the center. Finally, the true value of the combined effect is estimated based on the compensated funnel diagram. When our publication bias results show that there is publication bias, we can use cut and replacement to evaluate the impact of publication bias on the results; if the impact is not significant, it means that the authenticity of the results is better, and if the impact is relatively large (no statistical significance), it is necessary to fully discuss the impact of publication bias on the results.

### Prognostic analysis of TDO2 with TCGA data

We explored the relationship between TDO2 and prognosis and clinical features based on case–control or cohort studies in published articles. Usually, the expression of TDO2 at the protein level was detected by immunohistochemistry (IHC), and the expression of TDO2 at the RNA level was detected by a real-time quantitative PCR detection system (q-PCR). Sample size will generally be limited; thus, to further expand the sample size, we verified it through the TCGA database. We used the GEPIA2 website to search the TCGA database about TDO2 and clinicopathological features and survival and prognosis data. GEPIA2 is an online search website for tumor databases, which can query and analyze the RNA-seq expression data of tumor samples and normal samples (28). Five hundred similar genes with a correlation coefficient greater than 0.25 were selected from GEPIA2 and were significantly correlated with the TDO2 (**Table S3**). Furthermore, to study the potential biological function of TDO2 gene, we used a Web gene set analysis kit (WebGestalt) to analyze the pathway of Kyoto Encyclopedia of Genes and Genomes (KEGG) (29).

## Results

### Study selection

**Figure 1:**
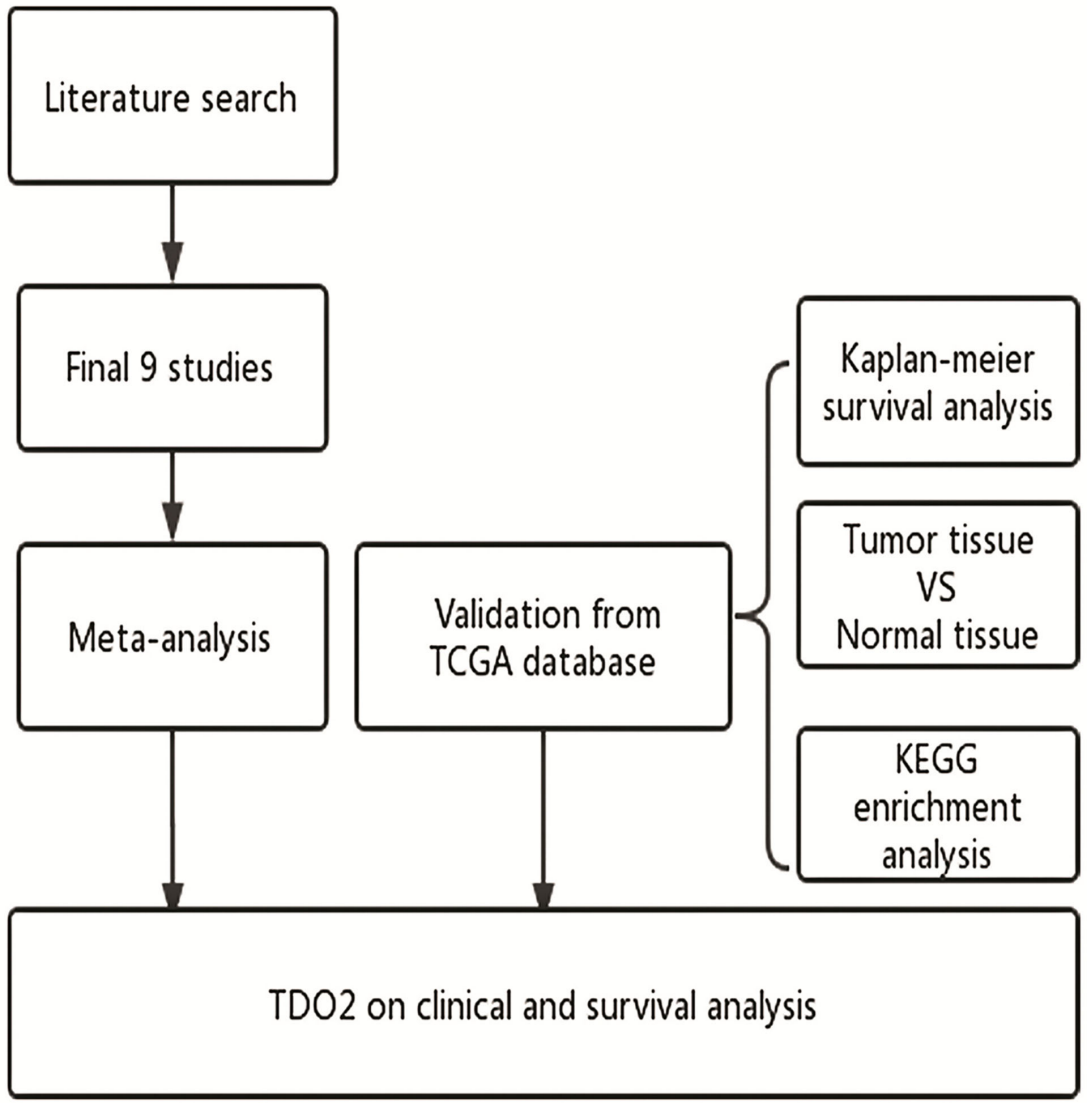
Flow diagram of the study. TDO2, tryptophan 2,3-dioxygenase; TCGA, The Cancer Genome Atlas; KEGG, Kyoto Encyclopedia of Genes and Genomes.

**Figure 2:**
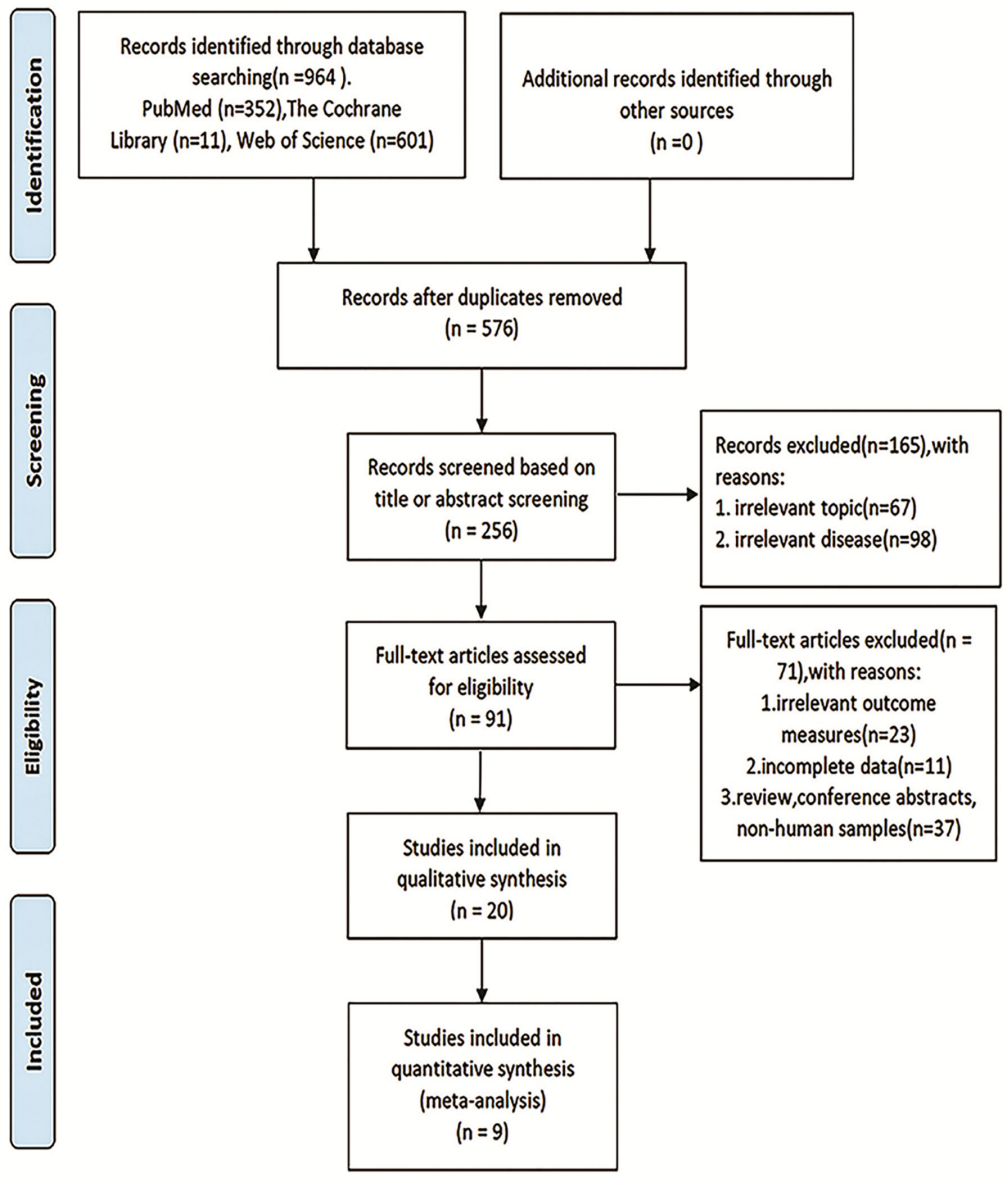
Flow diagram of the study identification, inclusion, and exclusion process.

The overall flowchart of this paper is shown in **Figure 1**, which is mainly composed of two parts: meta-analysis and bioinformatics verification. According to the PRISMA statement, the flowchart of the screening process for inclusion in the literature can be seen in **Figure 2**. According to the established retrieval strategy, a total of 964 articles were selected. In total, 388 articles were excluded because they were repetitive reports. After that, there are 567 articles excluded because of their titles, abstracts, and full texts. In the end, we included only nine articles that could be meta-analyzed to study the correlation between the level of TDO2 and survival outcomes in various malignant tumors (20, 30–37).

### Study characteristics

The literature we included was published from 2016 to 2021. Our meta-analysis included 667 patients, ranging from 31 to 192 patients in each study. Regarding the type of tumor, there were nine tumor types: esophageal carcinoma, colorectal cancer, hepatocellular carcinoma, Merkel cell carcinoma, soft tissue leiomyosarcoma, renal cell carcinoma, glioma, neuroendocrine tumor, and diffuse large B-cell lymphoma. Geographically, the original populations came from different countries; four articles were from China, four articles were from Japan, and one article pooled data from the Netherlands. The studies we included were all prospective cohort studies, and all studies were reported in English. It was divided into two parts: nine articles included the data on TDO2 expression and survival prognosis, six of which reported the data on clinicopathological features. The patients in these cohort studies were classified into two groups by the high or low level of TDO2. The basic data and information of the nine articles included in this meta-analysis are summarized in **Table 1**. In addition, we summarized the main IHC information in **Table 2**, including IHC antibody source, antibody dilution, antibody clone, and other major information.

**Table 1:** Main characteristics of the eligible studies for the meta-analysis.

No.	Author year (Ref. number)	Country	NOS score	Tumor types	Sample source	Number of patients	TDO2 expression		Outcome	Follow-up time	Method of detection	HR source
							High	Low				
1	Chen 2016 (35)	China	7	Colorectal cancer	Tumor tissue	192	88	104	OS, PFS	NA	IHC	KPM curve extraction
2	Chen 2020 (33)	China	8	Diffuse large B-cell lymphoma	Tumor tissue	60	30	30	OS, PFS	YES	IHC	KPM curve extraction
3	DU 2020 (31)	China	9	Glioma	Tumor tissue	41	17	24	OS	YES	IHC	KPM curve extraction
4	Hosson 2020 (32)	Netherlands	7	Neuroendocrine tumor	Tumor tissue	51	29	17	OS	NA	IHC	Direct reports
5	Li 2020 (36)	China	8	Hepatocellular carcinoma	Tumor tissue	93	77	16	OS, DFS	YES	IHC	KPM curve extraction
6	Lwasaki 2021 (20)	Japan	8	leiomyosarcoma of soft tissue	Tumor tissue	69	29	40	OS, PFS	YES	IHC	Direct reports
7	Pham 2018 (34)	Japan	8	Esophageal cancer	Tumor tissue	90	53	37	OS	YES	IHC	Direct reports
8	Sumitomo 2021 (30)	Japan	7	Renal cell carcinoma	Tumor tissue	40	15	25	OS, PFS	NA	IHC	KPM curve extraction
9	Wardhani 2019 (37)	Japan	7	Merkel cell carcinoma	Tumor tissue	31	10	21	OS, DSS	NA	IHC	Direct reports

Ref: reference; IHC, immunohistochemistry staining; TDO2, tryptophan 2,3-dioxygenase; OS, overall survival; DFS, disease-free survival; DSS, disease-free survival; PFS, progression-free survival; NA, not available; HR, hazard ratio; KPM, Kaplan–Meier survival curves.

**Table 2:** Summary of immunohistochemical methods for TDO2 detection in the studies included in this meta-analysis.

No.	Study	Detection method	Antibody source	Antibody dilution	Antibody clone	Antibody company	Cut-off value	Evaluation method
1	Chen2016(35)	IHC	NR	NR	NR	NR	>150 high expression	H score
2	Chen2020(33)	IHC	NR	NR	IDI	Abcam, Cambridge,U K	>5% positive	Fromvit composite score
3	DU2020(31)	IHC	Rabbit	1:50	NR	Abcam, UK	>93.29 high expression	H score
4	Hosson2020(32)	IHC	Rabbit	1:200	HPA039, 611	Atlas Antibodies, Bromma,Sweden	>1% positive	TME (%)
5	Li2020(36)	IHC	NR	1:500	NR	Novus,USA	negative0-3 weak4-6 strong8-12	staining area method
6	Lwasaki2021(20)	IHC	Mouse	1:100	OTI2A4	Lifespan Biosciences ,USA	≥50 high expression	H score
7	Pham2018(34)	IHC	Mouse	1:250	NR	Abnova, China	>55 high expression	H score
8	Sumitomo2021(30)	IHC	Mouse	1:100	4G2	OriGene Technologie,USA	1-2 low expression and 3-4 high expression	TDO2 expression level
9	Wardhani2019(37)	IHC	Mouse	1:100	NR	LifeSpan BioSciences, USA	≥101 or ≥64% high expression	modified H score or TME (%)

IHC, immunohistochemistry staining; NR, not reported; TDO2, tryptophan 2,3-dioxygenase; TME, tumor microenvironment.

### High-quality papers

According to the NOS, all the studies we included were of high quality, because the average score was 7.9, the lowest score was 7, the highest score was 9, and none of the studies scored less than 7. The details of the score can be summarized in **Table 3**.

**Table 3:** Newcastle–Ottawa Scale quality assessment of the enrolled studies.

No.	Study	Selection				Comparability		Outcome			Total
		Representativeness of the exposed cohort	Selection of the non-exposed cohort	Ascertainment of exposure	Outcome of interest	Control for factor (1)	Control for factor (2)	Assessment of outcome	Follow-up long enough	Adequacy of follow-up of cohorts	
1	Chen 2016 (35)	1	1	1	1	1	1	1	0	0	7
2	Chen 2020 (33)	1	1	1	1	1	1	1	1	0	8
3	DU 2020 (31)	1	1	1	1	1	1	1	1	1	9
4	Hosson 2020 (32)	1	1	1	1	1	1	1	0	0	7
5	Li 2020 (36)	1	1	1	1	1	1	1	1	0	8
6	Lwasaki 2021 (20)	1	1	1	1	1	1	1	1	0	8
7	Pham 2018 (34)	1	1	1	1	1	1	1	1	0	8
8	Sumitomo 2021 (30)	1	1	1	1	1	1	1	0	0	7
9	Wardhani 2019 (37)	1	1	1	1	1	1	1	0	0	7

### Associations between TDO2 expression and clinicopathological characteristics

Using the data included in the literature, this paper discussed the relationship between TDO2 and six clinicopathological features, including sex, TNM stage, and lymphatic metastasis. We found that overexpression of TDO2 was significantly related to the stage of lymph node metastasis (N0 versus N1, N2, N3; RR = 0.76, 95% CI = 0.59–0.99, *p* = 0.04) as well as TNM stage (I–II versus III–IV, RR = 0.65, 95% CI = 0.48–0.89, *p* = 0.002). We found no significant relationship between overexpression of TDO2 and sex (female versus male, RR = 1.04, 95% CI = 0.89–1.20, *p* = 0.72), age at diagnosis (younger versus elderly, RR = 1.12, 95% CI = 0.97–1.30, *p* = 0.12), T classification (T1 versus T2, T3, T4; RR = 0.90, 95% CI = 0.35–2.28, *p* = 0.82), or distant metastasis (M0 versus M1, RR = 0.71, 95% CI = 0.48–1.06, *p* = 0.09). The detailed data are shown in **Table 4**.

**Table 4:** Meta-analysis results of the associations of increased TDO2 expression with clinicopathological parameters.

Clinical features and prognosis	Studies (*n*)	RR/HR (95% CI)	*p*-value	Heterogenicity		Effects model
				*I*², %	*p-*value	
Age (younger *vs*. elderly)	7	1.12 [0.97, 1.30]	0.12	0	0.67	REM
Sex (female *vs*. male)	7	1.04 [0.89, 1.20]	0.72	0	0.71	REM
T classification (T1 *vs*. T2, T3, T4)	4	0.90 [0.35, 2.28]	0.82	68	0.02	REM
Lymph node metastasis (N0 *vs*. N1, N2, N3)	4	0.76 [0.59, 0.99]	0.04	15	0.32	REM
Distant metastasis (M0 *vs*. M1)	4	0.71 [0.48, 1.06]	0.09	27	0.25	REM
TNM stage (I–II *vs*. III–IV)	6	0.65 [0.48, 0.89]	0.002	49	0.08	REM
OS	9	2.58 [1.52, 4.40]	0.0005	60	0.01	REM
PFS	4	2.38 [0.99, 5.73]	0.05	85	0.0002	REM

TDO2, tryptophan 2,3-dioxygenase; RR, risk ratios; HR, hazard ratio; OS, overall survival; PFS, progression-free survival; CI, confidence interval; REM, random-effects model.

### Increased TDO2 expression and OS

**Figure 3:**
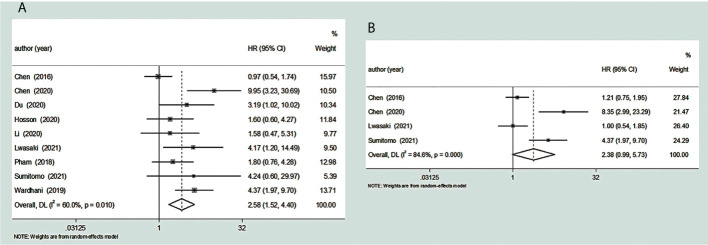
Forest plot for the relationship between TDO2 expression and OS/PFS. **(A)** The correlation between TDO2 expression and OS in human tumors. **(B)** The correlation between TDO2 expression and PFS in human tumors. HR, hazard ratio; 95% CI, 95% confidence interval; OS, overall survival; PFS, progression-free survival; TDO2, tryptophan 2,3-dioxygenase.

We included a cohort study of 667 patients with malignant tumors in nine articles to study the relationship between their TDO2 and OS. We found that there was high heterogeneity in the HR values after the combination of these studies (*I* ^2^ = 60.0%, *p* = 0.01). The data tell us that there is a significant connection between overexpression of TDO2 and poor OS (pooled HR = 2.58, 95% CI = 1.52–4.40, *p* = 0.0005) (**Figure 3**).

### Increased TDO2 expression and PFS

We included a total of four reports involving 361 patients with malignant tumors to study the correlation between their TDO2 and PFS. We found considerable heterogeneity between these studies (*I* ^2^ = 84.6%, *p* = 0.0002). The pooled HR was 2.38 (95% CI = 0.99–5.73, *p* = 0.05); these data showed that there is a close relationship between overexpressed TDO2 and poor PFS (**Figure 3**). Five papers did not have enough information to calculate the association between TDO2 and PFS in patients with malignant tumors; thus, they were not included (31, 32, 34, 36, 37).

### Subgroup analyses

To explain the source of heterogeneity of HR outcome between TDO2 and OS in patients with malignant tumors, the patients were classified according to their different potential factors such as cutoff value (H score or expression degree or other ways), sample size (sample size ≤ 70 or sample size >70), NOS score (score ≤ 7 or score >7), HR extraction source (direct reports or survival curve extraction), study country (Japan or China or other countries), and year of publication (<2019 or ≥2019). The subgroup analysis data are summarized in **Table 5**.

**Table 5:** **A** subgroup analysis was conducted to summarize studies of overall survival stratification according to different states.

Subgroups	Studies (*n*)	HR (95% CI)	*p*-value	Heterogeneity		Effects model
				*I*², %	*p*-value	
Overall survival	9	2.58 [1.52, 4.40]	0.0005	60	0.0005	REM
cutoff value						
H score	5	2.33 [1.19, 4.56]	0.01	65	0.02	REM
Expression degree	3	1.81 [0.89, 3.69]	0.1	0	0.66	REM
Other ways	1	9.95 [3.23, 30.69]	NA	NA	NA	REM
Sample size						
≤70	6	3.84 [2.34, 6.32]	<0.00001	17	0.3	REM
>70	3	1.23 [0.78, 1.92]	0.38	0	0.47	REM
NOS score						
≤7	4	2.05 [0.88, 4.78]	0.1	69	0.02	REM
>7	5	3.18 [1.65, 6.13]	0.0006	43	0.13	REM
HR source						
Direct reports	4	2.66 [1.56, 4.53]	0.0003	21	0.28	REM
Kp-m curve extraction	5	2.67 [1.03, 6.89]	0.04	73	0.005	REM
Country of study						
Japan	5	2.74 [1.74, 4.32]	<0.0001	0	0.4	REM
China	3	1.48 [0.73, 3.01]	0.28	42	0.18	REM
Other countries	1	9.95 [3.23, 30.69]	NA	NA	NA	REM
Published year						
<2019	2	1.21 [0.68, 2.16]	0.52	25	0.25	REM
≥2019	7	3.44 [2.11, 5.59]	0	23	0.25	REM

TDO2, tryptophan 2,3-dioxygenase; HR, hazard ratio; CI, confidence interval; Kp-m, Kaplan–Meier plotter; NA, not available; REM, random-effects model.

### Stratification by cutoff value

In the included literature, five articles used the H score (HR = 2.33, 95% CI = 1.19–4.56, *p* = 0.01). The remaining three articles used IHC expression degree (pooled HR = 1.81, 95% CI = 0.89–3.69, *p* = 0.1). Moreover, the last article reported that TDO2 expression evaluated using other ways was related to a worse OS (pooled HR = 9.95, 95% CI = 3.23–30.69).

### Stratified by sample size

When stratified by sample size, there was a statistically significant association between TDO2 overexpression and OS when sample size ≤70 (pooled HR = 3.84, 95% CI = 2.34–6.32, *p* < 0.00001). In contrast, when the sample size was >70, the association between TDO2 overexpression and OS was not statistically significant (pooled HR = 1.23, 95% CI = 0.78–1.92, *p* = 0.38).

### Stratified by NOS score

Stratified by NOS score, there was a significant association between TDO2 overexpression and poor OS when NOS score >7 was grouped (pooled HR = 3.18, 95% CI = 1.65–6.13, *p* = 0.0006). In contrast, when NOS score ≤7, there was no statistically significant association between TDO2 expression and OS (pooled HR = 2.05, 95% CI = 0.88–4.78, *p* = 0.1).

### Stratification by HR source

In terms of the HR source, TDO2 overexpression was significantly related to a worse prognosis in the direct reports group (pooled HR = 2.66, 95% CI = 1.56–4.53, *p* = 0.0003). The prognostic significance was also related to a worse OS prognosis in the Kaplan–Meier curve group (pooled HR = 2.67, 95% CI = 1.03–6.89, *p* = 0.04), and we did not consider the HR source to be a cause of the OS heterogeneity.

### Stratification by study country

The results for grouping by study country showed that TDO2 overexpression was related to poor survival outcomes in Japan (pooled HR = 2.74, 95% CI = 1.74–4.32, *p* < 0.0001) and The Netherlands (pooled HR = 9.95, 95% CI = 3.23–30.69). However, we found no obvious connection in China (pooled HR = 1.48, 95% CI = 0.73–3.01, *p* = 0.28).

### Stratification by year of publication

Subgroup analysis of publication year showed that there was a statistically significant connection between TDO2 expression and OS of articles published after 2019 (HR = 3.44, 95% CI = 2.11–5.59, *p* = 0.000). On the contrary, there was no statistical connection between TDO2 expression and OS before 2019 (HR = 1.21, 95% CI = 0.68–2.16, *p* = 0.52).

### Bioinformatic analysis

**Figure 4:**
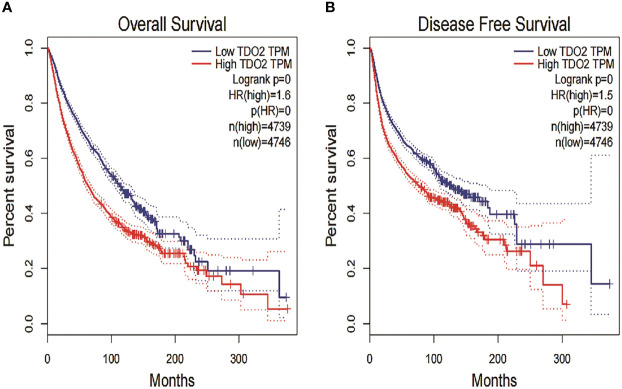
Kaplan–Meier plotter showed the prognostic role of high and low expression of TDO2 mRNA expression in OS/DFS. **(A)** OS in malignant tumor patients. **(B)** DFS in malignant tumor patients. HR, hazard ratio; CI, confidence interval; OS, overall survival; DFS, disease-free survival;TDO2, tryptophan 2,3-dioxygenase; TPM, transcripts per kilobase of exon model per million mapped reads.

**Figure 5:**
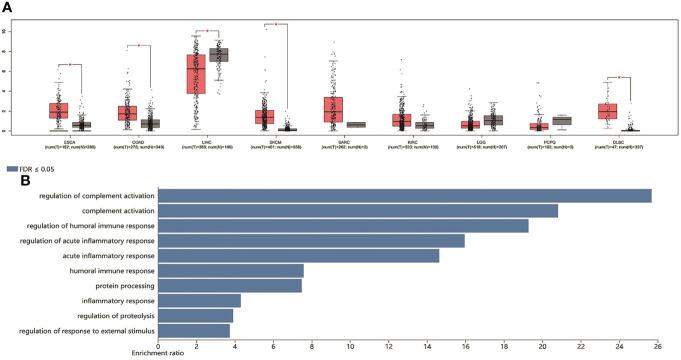
TDO2 expression in TCGA data set and biological function analysis. **(A)** The expression of TDO2 in malignant tumors and adjacent tissues **(B)** KEGG enrichment analysis of TDO2 related genes. TDO2, tryptophan 2,3-Dioxygenase; HR, hazard ratio; KEGG, Kyoto Encyclopedia of Genes and Genomes; FDR, false discovery rate; ESCA, esophageal carcinoma; COAD, colon adenocarcinoma; LIHC, liver hepatocellular carcinoma; SKCM, skin cutaneous melanoma; SARC, sarcoma; KIRC, kidney renal clear cell carcinoma; LGG, brain lower grade glioma; PCPG, pheochromocytoma and paraganglioma; DLBC, lymphoid neoplasm diffuse large B-cell lymphoma.*p-value < 0.05.

From the data of TCGA (*n* = 9,475 patients), we found that the high level of TDO2 was statistically significant in terms of poor OS (HR = 1.6, *p* < 0.001) (**Figure 4A**) and DFS (HR = 1.5, *p* < 0.001) (**Figure 4B**).We further verified the nine tumor types included in this paper. The comparative data of cancer and paracancerous tissues suggested that the expression of TDO2 in tumor tissue was higher than that in non-tumor tissue. As shown in **Figure 5A**, TDO2 was significantly upregulated in esophageal carcinoma (ESCA), colon adenocarcinoma (COAD), skin cutaneous melanoma (SKCM), sarcoma (SARC), kidney renal clear cell carcinoma (KIRC), and lymphoid neoplasm diffuse large B-cell lymphoma (DLBC) when compared with matched paracarcinoma tissues. Moreover, TDO2 may have a function in immune regulation. It was closely related to the regulation of complement activation, complement activation, regulation of the humoral immune response, regulation of the acute inflammatory response, the acute inflammatory response, the humoral immune response, and protein processing (**Figure 5B**).

### Sensitivity analysis

**Figure 6:**
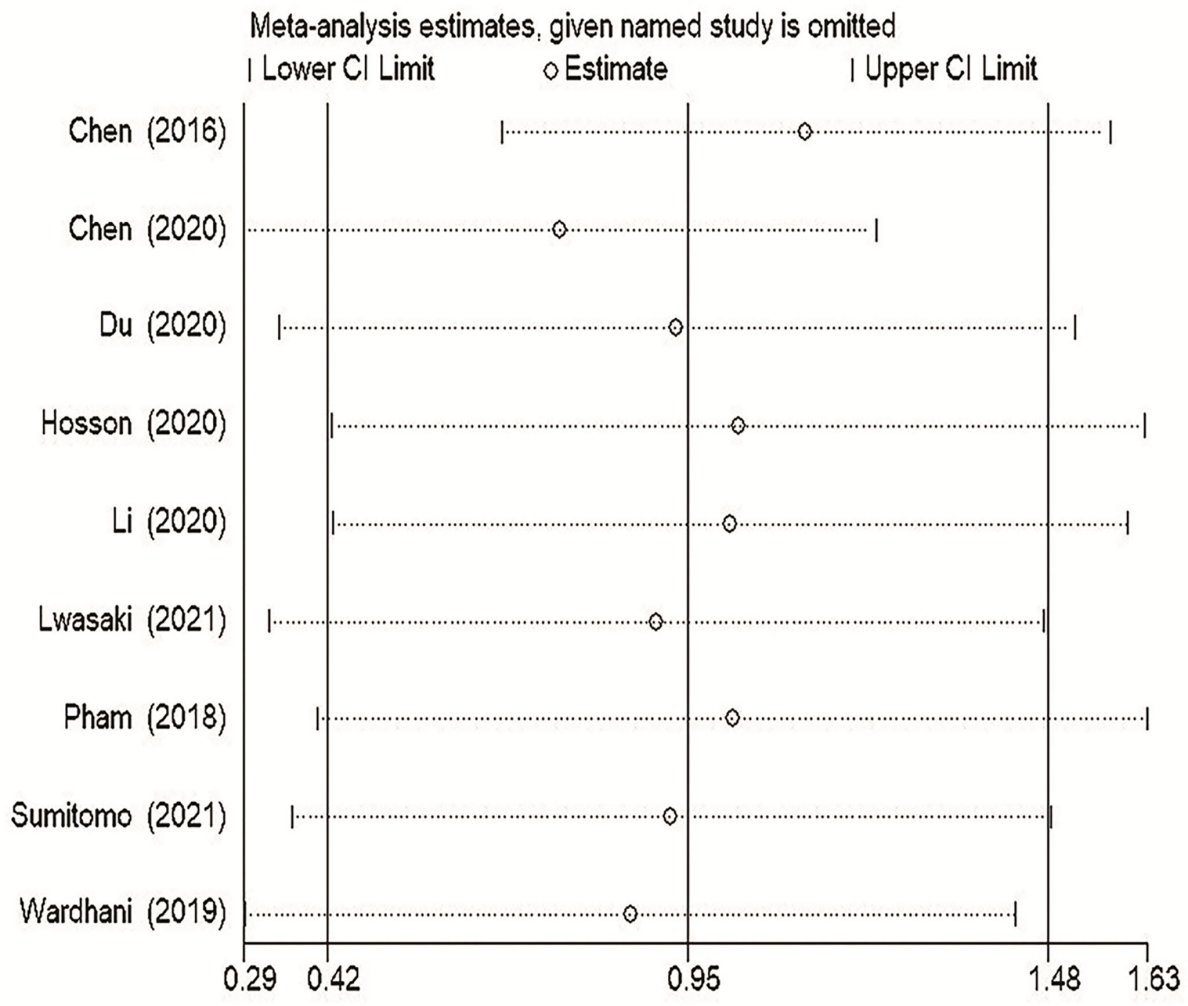
Sensitivity analysis of pooled HRs on the association between TDO2 expression and OS. OS, overall survival.

To explore the stability of our TDO2 between survival and prognosis OS results, we conducted a sensitivity analysis. In general, sensitivity analysis can not only evaluate the stability and reliability of the merger results of meta-analysis, but also evaluate whether the merger results have changed significantly due to the influence of a single study. As shown in **Figure 6**, after excluding any study, the upper and lower limits of the combined effect values did not fall outside the 95% confidence interval, and the impact of TDO2 expression on OS did not change significantly, indicating that our meta-analysis is robust and credible.

### Publication bias

**Figure 7:**
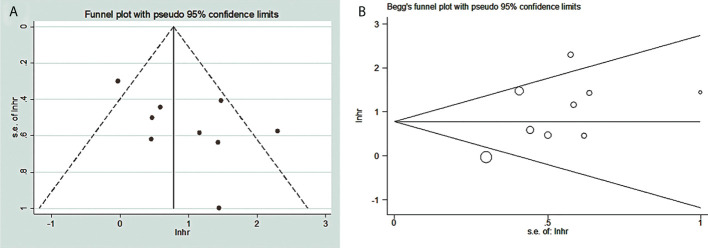
Publication bias between TDO2 and OS in patients with malignant tumors. **(A)** Funnel plots; **(B)** Begg’s test. TDO2, tryptophan 2,3-dioxygenase.

The funnel chart of publication bias shows that the amount of effect is the abscissa and the ordinate is standard error. “In” is converted to natural logarithm, “hr” is the effect index (HR) we selected, lnhr = ln(HR), lnhr is the natural logarithm of the hazard ratio. “s.e. of lnhr” is “selnhr”, which is the standard error of the natural logarithm of HR, and its calculation formula is selnhr = (lnul-lnll)/(1.96*2). ul and ll are the upper and lower bounds of the 95% confidence interval for HR, respectively. The dispersion of the small sample is larger, so it is often at the bottom of the funnel diagram, while the dispersion of the large sample is smaller, so it is at the top. Under normal circumstances, it should be small at the top and large at the bottom. If not, there may be a large bias. Our graphics are similar to this but somewhat asymmetrical, so there is a publication bias (**Figure 7**). In addition, our Begg test (*p* = 0.000) gives a specific *p*-value, which suggested that our results may have publication bias (**Figure 7**).

### Metatrim

**Figure 8:**
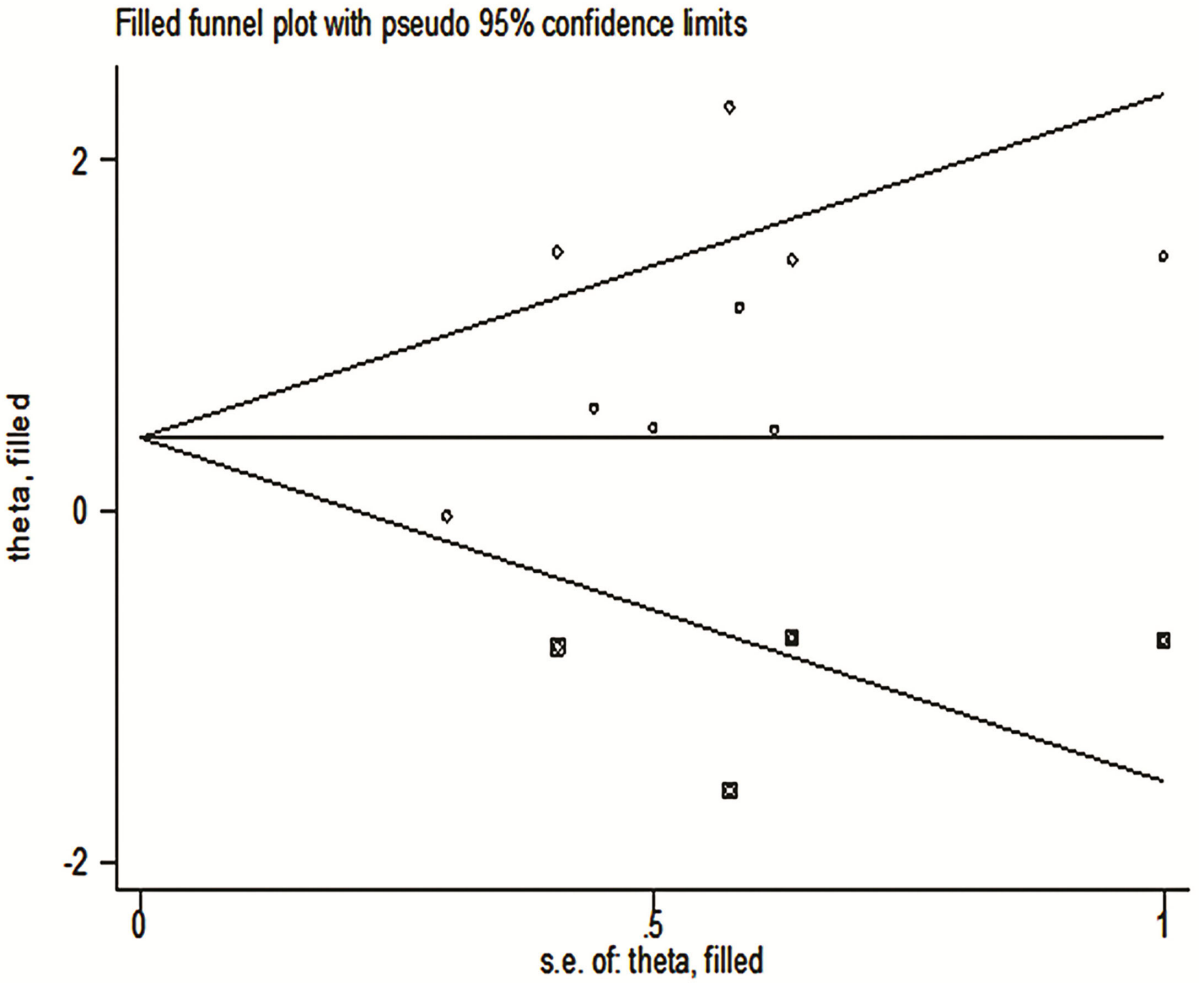
Trim-and-fill analysis.

Using trim-and-fill analysis, we found that there may be four studies that have not yet been published, and these four articles on the relationship between TDO2 and the survival outcome may not have been searched (**Figure 8**).The filled funnel plot ordinate is “theta”, and the abscissa is “s.e.of: theta”. “theta” is the target variable, and s.e.of:theta is the standard error of the target variable. The filled meta-analysis results for OS (HR = 1.42, 95% CI = 1.08–1.87, *p* = 0.012) was robust. The supplementary literature has little effect on the results of the original study, the results of trim and fill are the same as the results of the initial merger, there is a statistical difference, and publication bias does not affect the original results; hence, the results are reliable and credible.

## Discussion

TDO2 is a key enzyme that catalyzes the degradation of tryptophan in the Kyn pathway as the first and rate-limiting step (38, 39). Recent literature suggests that the Kyn signaling pathway negatively regulates the body’s immune response and is deeply involved in the occurrence and development of tumors. Therefore, inhibiting the activity of kynurenine-synthesis enzyme is considered an effective strategy to improve immunotherapy (40). Many kinds of malignant tumors use the TDO2 enzyme to promote the degradation and metabolism of tryptophan to avoid the destruction of their immunity (41). Overexpressed levels of TDO2 are found in many cancer cells, such as breast carcinoma, renal carcinoma, and melanoma (42–44). Therefore, these studies suggest that TDO2 should be an interesting therapeutic target.

TDO2 may be a prognostic marker and a promising therapeutic target for malignant cancers as suggested by a number of studies. These include TDO2 promoting the proliferation and invasion of liver cancer (36), promoting EMT in HCC by activating the Kyn-AhR pathway, thereby regulating HCC metastasis and invasion (45), and associating with MCC clinicopathological aspects and prognosis, with targeting the TDO-KYN-AhR pathway in combination with ICI therapy as a potential strategy against tumoral immune escape (37). TDO2 expression has also been implicated in renal cell carcinoma and as a prognostic biomarker for immunotolerance in mRCC patients (30) and in glioma cell motility by virtue of its correlation with aquaporin 4 expression (31). Activation of the KYN-AhR signaling pathway by TDO2 promotes tumoral immune escape and antitumor therapy response in malignancy (46), plays a key role in progression of glioblastoma multiforme (GBM) and in regulating GBM to induce cellular replication stress and DNA damage (47). TDO2 expression in most neural stem cells and CAFs can be used to explore antitumor therapy immune response (32).

Interestingly, correlation between TDO2 and the immune status of patients with malignant tumors is suggested by a number of studies. These include that fractions derived from cells with high TDO2 expression are capable of inhibiting T lymphopoiesis in other tumors. TDO2 overexpression in the tumor microenvironment leads to a decrease in T lymphocyte production, which, in turn, triggers immune escape and leads to a poor response to immunotherapy (48). In malignant patients with high TDO2 expression, negative regulation of the KYN-AHR pathway enhances the efficacy of indoleamine 2,3-dioxygenase 1 (IDO1) or TDO2 inhibitors and preferably in combination with ICIs such as PD1 (49). These findings suggest that TDO2 is undoubtedly an interesting target molecule for immunotherapy. Moreover, studies have also shown that TDO2 and IDO1 promote immunosuppression in patients with malignant tumors through the induction of bone marrow-derived suppressor cells (MDSCs) and through the interaction between Treg and macrophages (50). More interestingly, the expression of tryptophan is closely related to the development of many metabolic diseases besides tumors. It has been found that there may be a cellular interaction between the TDO2–AHR axis, which may induce an immune response from viruses in the respiratory tract. This evidence suggested that TDO2 expression was elevated in patients who develop abdominal fibrosis after bone marrow transplantation (BMT), and that elevated TDO2 subsequently produces large amounts of collagen. This study confirmed that TDO2 overexpression plays a key role in regulating the immune response against viruses (51). In addition to the above, TDO2 was also found to be overexpressed in patients with depression (52). Thus, TDO2 can be considered an interesting molecular target for the treatment of depressed patients. Apparently, TDO2 is a key factor in the TRP-KYN pathway, and it is also associated with immune response and psychiatric disease.

The expression of tryptophan metabolizing enzymes IDO1 and TDO2 has a strong correlation with the poor prognosis of various malignant tumors. Interestingly, in a recent study, elevated levels of tryptophan transporters (SLC1A5 and SLC7A5) were found to be significantly associated with decreased survival in renal cancer (53). Therefore, exploring the Kyn pathway may facilitate the development of new strategies for the diagnosis and treatment of renal cancer. IDO1 is also an enzyme as important as TDO2 in the KYN signaling pathway, and there have been three meta-analysis articles on the relationship between IDO1 and survival of patients with malignant tumors (54–56). However, a phase III clinical trial on IDO1 has been conducted, and the published survival data for IDO1 have lowered people’s expectations for this target (57). Some scholars said that although the results of IDO1 are not satisfactory, it was important to pay attention to the original aim of the clinical trials, which was to study how the catabolism of TRP is dysregulated and why it enables malignant tumors to obtain immune escape response and evade the overall efficiency of antitumor therapy (49). There have been many articles on the role of IDO1 in antitumor therapy immunity (13, 58, 59). However, alternative approaches targeting TDO2 for tumor immunotherapy have been neglected. Therefore, some articles also analyze whether combination therapy additionally targeting TDO2 would be effective (60). In summary, our study investigated the overexpression of the immune checkpoint molecule TDO2 and its importance in malignant tumors to provide a basis for the development of drugs or inhibitors targeting TDO2.

Through molecular docking technology, the binding mode of compound 9O-23 within the TDO2 binding pocket has been predicted. Meanwhile, compound 9O-23 presented a strong inhibitory effect on TDO2 in enzymology and therefore promoted the proliferation of T cells, which optimized the structure of existing TDO2 inhibitors (61). Compound F04 was found to enhance the inhibitory activity of TDO2 by molecular docking technology, indicating that F04 is a promising inhibitor of TDO2 (62). In addition, an optimized compound 21 as potent TDO2 inhibitor was identified from a high-throughput screen (HTS). This optimized compound 21 is a potent TDO2 inhibitor with modest selectivity over IDO1 and improved its stability in the blood (63). In conclusion, many compounds that can effectively inhibit TDO2 have been identified by performing virtual binding methods through molecular docking technology or molecular dynamics simulation, providing favorable conditions to further optimize the structure of TDO2 inhibitors, also for the further development of selective inhibitors and dual inhibitors.

Our paper is the first meta-analysis to comprehensively study the survival and prognostic value of TDO2 in patients with malignant tumors. The final data demonstrated that overexpression of TDO2 was significantly related to worse survival outcomes. For OS, HR indicated a strong correlation between overexpression of TDO2 and poor OS (HR = 2.58, 95% CI = 1.52–4.40, *p* = 0.0005), suggesting that TDO2 overexpression is a bad factor for patients with malignant tumor. For PFS, HR showed that overexpression of TDO2 was correlated with poor PFS (HR = 2.38, 95% CI = 0.99–5.73, *p* = 0.05), although the result was not statistically significant. Furthermore, the final data showed that overexpression of TDO2 was significantly related to regional lymph node metastasis (RR = 0.76, 95% CI = 0.59–0.99, *p* = 0.04) and TNM stage (RR = 0.65, 95% CI = 0.48–0.89, *p* = 0.002). We found no statistically significant relationship between TDO2 overexpression and sex, age at diagnosis, T classification, or distant metastasis.

We included a total of nine studies; for each study, we conducted a NOS score, and the overall score results tell us that the bias between the included studies is very small. It can be called high-quality research. For overexpressed TDO2 and worse OS survival outcomes, we conducted a sensitivity analysis, and we found that excluding each study, the combined results were still statistically significant, indicating that our results are robust and reliable. In addition, we also tested the publication bias. From the subjective funnel chart, we can find that the icon is asymmetric and there is publication bias. Our visual subjective judgment is verified in the qualitative Begg analysis data. It showed that there is publication bias in the study included in our meta-analysis. To investigate this bias, we have carried out the method of clipping and compensation, and the conclusion is that there are four potential studies. They may exist in unsearched negative databases or future unpublished studies, and the combined HR results of these four studies are statistically significant, which further indicates that our results are robust.

More importantly, for the discussion of heterogeneity, we have fully carried out a subgroup analysis, which is of great help to the promotion of our research results. After screening all contents, we thought that the cutoff value, sample size, NOS score, study country, and year of publication were the causes of high heterogeneity. Our subgroup analysis demonstrated that overexpression of TDO2 was significantly related to poor OS. Stratified analysis by study country indicated that TDO2 overexpression was related to a worse OS for Europeans (HR = 9.95, 95% CI = 3.23–30.69, *p* < 0.05) and Japanese (HR = 2.74, 95% CI = 1.74–4.32), but there was no significant statistical significance for Chinese (HR = 1.48, 95% CI = 0.73–3.01, *p* = 0.28). The results of this subgroup analysis should be viewed with caution, as there are many variables between different countries, such as lifestyle, eating habits, smoking, drinking, diagnostic criteria, and primary care facilities. Any conclusions in this regard need to await cross-country comparative studies. Stratification by cutoff value indicated significant correlation between TDO2 overexpression and OS in H score (HR = 2.33, 95% CI = 1.19–4.56, *p* = 0.01), expression degree (HR = 1.81, 95% CI = 0.89–3.69, *p* = 0.1), and other ways (HR = 9.95, 95% CI = 3.23–30.69, *p* < 0.05). Stratifying by NOS score, TDO2 overexpression showed a negative prognostic impact on OS in NOS score >7 (HR = 3.18, 95% CI = 1.65–6.13, *p* = 0.0006) and score ≤7 (HR = 2.05, 95% CI = 0.88–4.78, *p* = 0.1). Stratified analysis by year of publication demonstrated that TDO2 overexpression was linked to an unfavorable OS after 2019 (HR = 3.44, 95% CI = 2.11–5.59, *p* = 0.000), and before 2019 (HR = 1.21, 95% CI = 0.68–2.16, *p* = 0.52) also showed a worse OS. We can see worse prognosis for patients after 2019. Experts note that standards of care and diagnosis generally improve over time. Why do these differences occur? A simple explanation may be due to the larger number of patients after 2019 (385 cases) than before 2019 (282 cases). Therefore, more case information is needed in future studies to conduct these studies. Stratifying by HR source, TDO2 overexpression was linked to worse prognosis for OS by the direct reports group (HR =2.66, 95% CI = 1.56–4.53, *p* = 0.0003) and the Kaplan–Meier curve group (HR = 2.67, 95% CI = 1.03–6.89, *p* = 0.04). When stratified by sample size, TDO2 overexpression was not significantly correlated with worse OS in the group of sample size >70 (HR = 1.23, 95% CI = 0.78–1.92, *p* = 0.38); however, it was related to worse OS in the group of sample size ≤70 (HR = 3.84, 95% CI = 2.34–6.32, *p* < 0.00001). In general, evidence from a larger sample size should be more convincing. We consider that the overall sample size included is not large; thus, we expect more rigorous and large-scale randomized controlled trials (RCTs) to provide more conclusive evidence.

Data analysis from the TCGA database further confirmed the prognostic value of TDO2 in malignant tumors. We used the GEPIA2 website to study the relationship between TDO2 expression in the TCGA database and the survival prognosis of patients with malignant tumor. Kaplan−Meier Plotter analysis showed that TDO2 overexpression was strongly related to poorer OS in 9,475 cancer patients (HR = 1.6, *p* < 0.001), and TDO2 overexpression was related to worse DFS in 9,475 cancer patients (HR = 1.5, *p* < 0.001). TCGA data sets showed that TDO2 was elevated at the mRNA level in most malignant tumors. Survival analysis of TCGA data showed that the survival rate of overexpressed TDO2 was much lower than that of underexpressed TDO2. Moreover, the expression of TDO2 was high in malignant tumors and higher in cancer tissue than in matched paracarcinoma tissue, which indicated that the expression of TDO2 was significantly associated with an unfavorable prognosis. We performed KEGG analysis of TDO2-related genes, and the final results showed that TDO2 was involved in a variety of immune responses, including regulation of complement activation, complement activation, regulation of the humoral immune response, regulation of the acute inflammatory response, the acute inflammatory response, the humoral immune response, and protein processing. These results suggest that TDO2 has a vital role in immune regulation in cancer. These analyses suggest that TDO2 may serve as a biomarker of worse prognosis in patients with malignant tumors. In addition, drug development targeting TDO2 is a potential tumor immunotherapy approach. The prognostic value of TDO2 in malignant tumors has been demonstrated in several different cohorts; however, confirmation of the prognostic value of TDO2 in different malignant tumors requires studies in larger cohorts of patients and cancer types.

### Limitations and advantages

Three limitations, however, should be taken into account when the findings of the present meta-analysis are interpreted. First, only English literature was retrieved, which may lead to incomplete retrieval and linguistic bias. Second, most of the patients in the study were from Japan and China, and there was a lack of sources from other regions. In other words, most studies (*n* = 8) used Asian patients, and the remaining one (*n* = 1) used European patients, who were geographically underrepresented. However, the above-mentioned limitations notwithstanding the present study found that TDO2 can serve as a promising prognostic marker and a potential immunotherapy target in malignant tumors. First, before the start of the research, we registered this study with the Cochrane collaboration network, and referenced the PRISMA statement to guide our writing. Second, this is the first meta-analysis of the relationship between TDO2 and the prognosis of patients with malignant tumors. In addition, the results of this study are robust due to the strict inclusion criteria. At the same time, over 9,000 cancer patients from TCGA data were also included to confirm our results. Third, we performed a subgroup analysis of the outcome of the study to explain the source of heterogeneity, providing potential help for the stratification of patients in larger clinical studies in the future, and also providing a reference for the application of TDO2 in different types of malignant tumors. Finally, we conducted a rigorous methodological evaluation of conclusions obtained from the present meta-analysis to ensure the credibility and quality of the research. Although there are some shortcomings in our study, our study showed that overexpression of TDO2 is related to worse survival outcomes. In the process of judging the survival and prognosis of patients and determining the treatment plan, doctors can evaluate the TDO2 level of patients with malignant tumors, to help reasonably and effectively stratify the clinical and therapeutic strategy of patients.

### Future direction

There are a lot of things that we need to focus on in future research. Firstly, high and low TDO2 expression should be defined based on a standard; therefore, the detection methods and defining standards of high and low TDO2 expression levels should be explored. Secondly, more studies are needed to verify the mechanism of TDO2 mediating tumor genesis and development. Furthermore, more molecules that may be associated with survival markers of malignant tumors such as tryptophan-related genes or Trp/Kyn ratio should be explored to help predict the prognosis of patients. Depriving tumors of essential nutrients, such as amino acids, is a promising cancer treatment strategy (64). Therefore, TDO2 inhibition may be a useful therapeutic intervention for cancers. In addition to using TDO2 inhibitors individually, TDO2 inhibitors are usually used in combination with IDO1 or other ICIs (13), in combination with glutathione S-transferase inhibitors (65), in combination with the immunomodulatory vaccine (66, 67), and in combination with radiotherapy and chemotherapy (67, 68). Moreover, in other diseases related to the KYN signaling pathway, TDO2 can also be used as a potential target for therapy (69, 70). Therefore TDO2 is of great significance not only for cancer, but also for neurological diseases (71), such as Alzheimer’s disease (AD) and Huntington’s disease (72). It also plays a critical role in treating depression (73). TDO2 has even been reported to inhibit *Staphylococcus aureus* and chlamydia (69). In general, TDO2 has a bright future and needs to be validated with a larger sample size and a more rigorous design.

## Conclusion

Our meta-study shows that the overexpression of TDO2 is closely related to the relatively poor survival prognosis and may become a potential biomarker for the prognosis of malignant tumors. Our biological mechanism analysis also shows that TDO2 plays a key role in the immune response. The immune checkpoint molecule TDO2 may be a valuable target for immunotherapy. It is expected that large-scale clinical studies need to verify this result.

## Data availability statement

The original contributions presented in the study are included in the article/**Supplementary Material**. Further inquiries can be directed to the corresponding author.

## Author contributions

YH and ZL collected, extracted, and performed quality assessment of articles. YH analyzed the data. YH and HT conceived and designed this study. YH wrote the paper. HT reviewed and revised the manuscript. All authors contributed to the article and approved the submitted version.

## Funding

This meta-analysis is supported by the National Natural Science Foundation of China [No.82260483 and No.81460463], the Joint Yunnan Provincial Foundation of Science and Technology Department and Kunming Medical University [2019FE001 (–173)], and the Yunnan Digestive Endoscopy Clinical Medical Center Foundation for Health Commission of Yunnan Province [2X2019-01-02].

## Conflict of interest

The authors declare that the research was conducted in the absence of any commercial or financial relationships that could be construed as a potential conflict of interest.

## Publisher’s note

All claims expressed in this article are solely those of the authors and do not necessarily represent those of their affiliated organizations, or those of the publisher, the editors and the reviewers. Any product that may be evaluated in this article, or claim that may be made by its manufacturer, is not guaranteed or endorsed by the publisher.

## Supplementary material

Supplemental material S1 PRISMA checklist. **S2.** A complete electronic search strategy for the PubMed database **S3.** Similar genes associated with TDO2.

## Abbreviations

TDO2, tryptophan 2,3-dioxygenase; Trp, tryptophan; Kyn, kynurenine; PRISMA, Preferred Reporting Items for Systematic Reviews and Meta-analysis; NOS, Newcastle–Ottawa Scale; RRs, risk ratios; HRs, hazard ratios; OS, overall survival; PFS, progression-free survival; TCGA, The Cancer Genome Atlas; TNM, tumor–node–metastasis; ICIs, immune checkpoint inhibitors; PROSPERO, International prospective register of systematic reviews; DFS, disease-free survival; q-PCR, real-time quantitative PCR detection system; IHC, immunohistochemistry; GEPIA2, Gene Expression Profiling Interactive Analysis; WebGestalt, Web gene set analysis kit; KEGG, Kyoto Encyclopedia of Genes and Genomes; EMT, epithelial-to-mesenchymal transition; AHR, aryl hydrocarbon receptor; mRCC, metastatic renal cell carcinoma; AQP4, aquaporin 4; CAFs, cancer-associated fibroblasts; GBM, glioblastoma multiforme; DNA, deoxyribonucleic acid; IDO1, indoleamine 2,3-dioxygenase 1; MDSCs, marrow-derived suppressor cells; BMT, bone marrow transplantation; HTS, high-throughput screen; AD, Alzheimer’s disease.
