# Supplementary material for: Tryptophan 2,3-dioxygenase may be a potential prognostic biomarker and immunotherapy target in cancer: A meta-analysis and bioinformatics analysis
Source: Front Oncol. 2022 Oct 3;12:977640. doi: 10.3389/fonc.2022.977640 (PMC9574363; doi:10.3389/fonc.2022.977640)
Supplement: Supplementary file 1 [file Table_2.doc]

Search: **（Tryptophan 2,3-Dioxygenase OR TDO2）and（prognosis OR survival OR outcome OR prognostic ）and（cancer OR tumor OR neoplasm OR carcinoma）** Sort by: **Publication Date**

("tryptophanase"[MeSH Terms] OR "tryptophanase"[All Fields] OR ("tryptophan"[All Fields] AND "2 3"[All Fields] AND "dioxygenase"[All Fields]) OR "tryptophan 2 3 dioxygenase"[All Fields] OR "tryptophan oxygenase"[MeSH Terms] OR ("tryptophan"[All Fields] AND "oxygenase"[All Fields]) OR "tryptophan oxygenase"[All Fields] OR ("tryptophan"[All Fields] AND "2 3"[All Fields] AND "dioxygenase"[All Fields]) OR "TDO2"[All Fields]) AND ("prognosis"[MeSH Terms] OR "prognosis"[All Fields] OR "prognoses"[All Fields] OR ("mortality"[MeSH Subheading] OR "mortality"[All Fields] OR "survival"[All Fields] OR "survival"[MeSH Terms] OR "survivability"[All Fields] OR "survivable"[All Fields] OR "survivals"[All Fields] OR "survive"[All Fields] OR "survived"[All Fields] OR "survives"[All Fields] OR "surviving"[All Fields]) OR ("outcome"[All Fields] OR "outcomes"[All Fields]) OR ("prognostic"[All Fields] OR "prognostical"[All Fields] OR "prognostically"[All Fields] OR "prognosticate"[All Fields] OR "prognosticated"[All Fields] OR "prognosticates"[All Fields] OR "prognosticating"[All Fields] OR "prognostication"[All Fields] OR "prognostications"[All Fields] OR "prognosticator"[All Fields] OR "prognosticators"[All Fields] OR "prognostics"[All Fields])) AND ("cancer s"[All Fields] OR "cancerated"[All Fields] OR "canceration"[All Fields] OR "cancerization"[All Fields] OR "cancerized"[All Fields] OR "cancerous"[All Fields] OR "neoplasms"[MeSH Terms] OR "neoplasms"[All Fields] OR "cancer"[All Fields] OR "cancers"[All Fields] OR ("cysts"[MeSH Terms] OR "cysts"[All Fields] OR "cyst"[All Fields] OR "neurofibroma"[MeSH Terms] OR "neurofibroma"[All Fields] OR "neurofibromas"[All Fields] OR "tumor s"[All Fields] OR "tumoral"[All Fields] OR "tumorous"[All Fields] OR "tumour"[All Fields] OR "neoplasms"[MeSH Terms] OR "neoplasms"[All Fields] OR "tumor"[All Fields] OR "tumour s"[All Fields] OR "tumoural"[All Fields] OR "tumourous"[All Fields] OR "tumours"[All Fields] OR "tumors"[All Fields]) OR ("neoplasm s"[All Fields] OR "neoplasms"[MeSH Terms] OR "neoplasms"[All Fields] OR "neoplasm"[All Fields]) OR ("carcinoma"[MeSH Terms] OR "carcinoma"[All Fields] OR "carcinomas"[All Fields] OR "carcinoma s"[All Fields]))

**Translations**

**Tryptophan 2,3-Dioxygenase:** "tryptophanase"[MeSH Terms] OR "tryptophanase"[All Fields] OR ("tryptophan"[All Fields] AND "2,3"[All Fields] AND "dioxygenase"[All Fields]) OR "tryptophan 2,3 dioxygenase"[All Fields] OR "tryptophan oxygenase"[MeSH Terms] OR ("tryptophan"[All Fields] AND "oxygenase"[All Fields]) OR "tryptophan oxygenase"[All Fields] OR ("tryptophan"[All Fields] AND "2,3"[All Fields] AND "dioxygenase"[All Fields])

**prognosis:** "prognosis"[MeSH Terms] OR "prognosis"[All Fields] OR "prognoses"[All Fields]

**survival:** "mortality"[Subheading] OR "mortality"[All Fields] OR "survival"[All Fields] OR "survival"[MeSH Terms] OR "survivability"[All Fields] OR "survivable"[All Fields] OR "survivals"[All Fields] OR "survive"[All Fields] OR "survived"[All Fields] OR "survives"[All Fields] OR "surviving"[All Fields]

**outcome:** "outcome"[All Fields] OR "outcomes"[All Fields]

**prognostic:** "prognostic"[All Fields] OR "prognostical"[All Fields] OR "prognostically"[All Fields] OR "prognosticate"[All Fields] OR "prognosticated"[All Fields] OR "prognosticates"[All Fields] OR "prognosticating"[All Fields] OR "prognostication"[All Fields] OR "prognostications"[All Fields] OR "prognosticator"[All Fields] OR "prognosticators"[All Fields] OR "prognostics"[All Fields]

**cancer:** "cancer's"[All Fields] OR "cancerated"[All Fields] OR "canceration"[All Fields] OR "cancerization"[All Fields] OR "cancerized"[All Fields] OR "cancerous"[All Fields] OR "neoplasms"[MeSH Terms] OR "neoplasms"[All Fields] OR "cancer"[All Fields] OR "cancers"[All Fields]

**tumor:** "cysts"[MeSH Terms] OR "cysts"[All Fields] OR "cyst"[All Fields] OR "neurofibroma"[MeSH Terms] OR "neurofibroma"[All Fields] OR "neurofibromas"[All Fields] OR "tumor's"[All Fields] OR "tumoral"[All Fields] OR "tumorous"[All Fields] OR "tumour"[All Fields] OR "neoplasms"[MeSH Terms] OR "neoplasms"[All Fields] OR "tumor"[All Fields] OR "tumour's"[All Fields] OR "tumoural"[All Fields] OR "tumourous"[All Fields] OR "tumours"[All Fields] OR "tumors"[All Fields]

**neoplasm:** "neoplasm's"[All Fields] OR "neoplasms"[MeSH Terms] OR "neoplasms"[All Fields] OR "neoplasm"[All Fields]

**carcinoma:** "carcinoma"[MeSH Terms] OR "carcinoma"[All Fields] OR "carcinomas"[All Fields] OR "carcinoma's"[All Fields]
