# Supplementary material for: Tryptophan 2,3-dioxygenase may be a potential prognostic biomarker and immunotherapy target in cancer: A meta-analysis and bioinformatics analysis
Source: Front Oncol. 2022 Oct 3;12:977640. doi: 10.3389/fonc.2022.977640 (PMC9574363; doi:10.3389/fonc.2022.977640)
Supplement: Supplementary file 2 [file Table_3.doc]

| Gene Symbol | Gene ID |
| --- | --- |
| AGXT | ENSG00000172482.4 |
| PLGLB2 | ENSG00000125551.18 |
| F2 | ENSG00000180210.14 |
| PLGLB1 | ENSG00000183281.14 |
| SERPINA10 | ENSG00000140093.9 |
| ARG1 | ENSG00000118520.13 |
| HPX | ENSG00000110169.10 |
| ALB | ENSG00000163631.16 |
| C8B | ENSG00000021852.12 |
| CPN2 | ENSG00000178772.6 |
| RBP4 | ENSG00000138207.12 |
| F13B | ENSG00000143278.3 |
| AMBP | ENSG00000106927.11 |
| APOH | ENSG00000091583.10 |
| GC | ENSG00000145321.12 |
| F11 | ENSG00000088926.13 |
| PLGLA | ENSG00000240935.6 |
| MAT1A | ENSG00000151224.12 |
| VTN | ENSG00000109072.13 |
| HRG | ENSG00000113905.4 |
| RP5-1103B4.3 | ENSG00000236341.1 |
| APOF | ENSG00000175336.9 |
| TFR2 | ENSG00000106327.12 |
| SLC38A4 | ENSG00000139209.15 |
| GBP7 | ENSG00000213512.1 |
| ITIH4 | ENSG00000055955.15 |
| F12 | ENSG00000131187.9 |
| SAA4 | ENSG00000148965.8 |
| ABCG8 | ENSG00000143921.6 |
| CPB2 | ENSG00000080618.13 |
| KNG1 | ENSG00000113889.11 |
| HAO1 | ENSG00000101323.4 |
| APOB | ENSG00000084674.13 |
| GLYCTK | ENSG00000168237.17 |
| APOC3 | ENSG00000110245.11 |
| APOC4-APOC2 | ENSG00000224916.8 |
| ORM2 | ENSG00000228278.3 |
| AC009963.3 | ENSG00000233494.1 |
| PON1 | ENSG00000005421.8 |
| C8A | ENSG00000157131.10 |
| KLKB1 | ENSG00000164344.15 |
| SERPINA4 | ENSG00000100665.11 |
| FGB | ENSG00000171564.11 |
| ABCG5 | ENSG00000138075.11 |
| PIPOX | ENSG00000179761.11 |
| SLC22A25 | ENSG00000196600.12 |
| SERPINC1 | ENSG00000117601.13 |
| RP11-116D2.1 | ENSG00000261012.2 |
| CYP2C8 | ENSG00000138115.13 |
| RP5-849H19.3 | ENSG00000278967.1 |
| ACOT12 | ENSG00000172497.8 |
| C3P1 | ENSG00000167798.16 |
| AMDHD1 | ENSG00000139344.7 |
| RP11-959F10.6 | ENSG00000256863.1 |
| FGA | ENSG00000171560.14 |
| INHBC | ENSG00000175189.3 |
| ANGPTL3 | ENSG00000132855.4 |
| UGT2B4 | ENSG00000156096.12 |
| GCKR | ENSG00000084734.8 |
| SARDH | ENSG00000123453.16 |
| PLG | ENSG00000122194.18 |
| CFH | ENSG00000000971.15 |
| UPB1 | ENSG00000100024.14 |
| SLC38A3 | ENSG00000188338.14 |
| GYS2 | ENSG00000111713.2 |
| APOA5 | ENSG00000110243.11 |
| SLCO1B1 | ENSG00000134538.2 |
| SERPIND1 | ENSG00000099937.10 |
| C6 | ENSG00000039537.13 |
| HSD17B6 | ENSG00000025423.11 |
| PCK2 | ENSG00000100889.11 |
| CYP4F2 | ENSG00000186115.12 |
| ORM1 | ENSG00000229314.5 |
| ITIH1 | ENSG00000055957.10 |
| LRG1 | ENSG00000171236.9 |
| C8G | ENSG00000176919.11 |
| AFM | ENSG00000079557.4 |
| LINC01485 | ENSG00000254211.5 |
| CFHR2 | ENSG00000276911.1 |
| PROZ | ENSG00000126231.13 |
| F9 | ENSG00000101981.10 |
| LINC01146 | ENSG00000258867.5 |
| ITIH3 | ENSG00000162267.12 |
| ALDOB | ENSG00000136872.17 |
| UGT2B15 | ENSG00000196620.8 |
| TAT | ENSG00000198650.10 |
| CFHR1 | ENSG00000244414.6 |
| C5 | ENSG00000106804.7 |
| OTC | ENSG00000036473.6 |
| HRSP12 | ENSG00000132541.10 |
| FGG | ENSG00000171557.16 |
| PROC | ENSG00000115718.17 |
| MTTP | ENSG00000138823.12 |
| AKR1D1 | ENSG00000122787.14 |
| C2 | ENSG00000166278.14 |
| HAL | ENSG00000084110.10 |
| F7 | ENSG00000057593.13 |
| PXMP2 | ENSG00000176894.9 |
| ASGR2 | ENSG00000161944.16 |
| C4BPB | ENSG00000123843.12 |
| A1CF | ENSG00000148584.14 |
| PFKFB1 | ENSG00000158571.10 |
| SLC10A1 | ENSG00000100652.4 |
| CREB3L3 | ENSG00000060566.13 |
| ADH1A | ENSG00000187758.7 |
| HP | ENSG00000257017.8 |
| RP11-372E1.4 | ENSG00000243818.4 |
| RP11-256L6.3 | ENSG00000257878.1 |
| RP11-344P13.4 | ENSG00000228826.2 |
| PAQR9-AS1 | ENSG00000241570.8 |
| GLS2 | ENSG00000135423.12 |
| CCND2P1 | ENSG00000256847.1 |
| ASGR1 | ENSG00000141505.11 |
| ANG | ENSG00000214274.9 |
| SERPINF2 | ENSG00000167711.13 |
| GPT | ENSG00000167701.13 |
| CYP2C9 | ENSG00000138109.9 |
| ASL | ENSG00000126522.16 |
| MASP2 | ENSG00000009724.16 |
| SERPINA1 | ENSG00000197249.12 |
| ITIH4-AS1 | ENSG00000239799.1 |
| APOC1 | ENSG00000130208.9 |
| SDS | ENSG00000135094.10 |
| RP11-108O10.2 | ENSG00000254990.5 |
| APOA1 | ENSG00000118137.9 |
| LINC01430 | ENSG00000237907.1 |
| SLC13A5 | ENSG00000141485.15 |
| SERPING1 | ENSG00000149131.15 |
| RP11-622A1.2 | ENSG00000250436.1 |
| SLC2A2 | ENSG00000163581.13 |
| CYP4A22 | ENSG00000162365.11 |
| AKR1C6P | ENSG00000151631.8 |
| LEAP2 | ENSG00000164406.7 |
| MTHFS | ENSG00000136371.9 |
| LINC01018 | ENSG00000250056.5 |
| AP006216.5 | ENSG00000236267.1 |
| UGT2B10 | ENSG00000109181.11 |
| TMEM56 | ENSG00000152078.9 |
| APOC2 | ENSG00000234906.8 |
| TF | ENSG00000091513.14 |
| PAH | ENSG00000171759.8 |
| RP5-966M1.6 | ENSG00000243696.4 |
| CFHR2 | ENSG00000080910.11 |
| INSIG1 | ENSG00000186480.12 |
| CYP2C18 | ENSG00000108242.12 |
| SLC22A7 | ENSG00000137204.14 |
| RP11-1151B14.2 | ENSG00000267675.1 |
| CYP2B6 | ENSG00000197408.8 |
| MST1 | ENSG00000173531.15 |
| DAO | ENSG00000110887.7 |
| NR1I3 | ENSG00000143257.11 |
| HAAO | ENSG00000162882.14 |
| MLXIPL | ENSG00000009950.15 |
| FGGY | ENSG00000172456.16 |
| SERPINA7 | ENSG00000123561.14 |
| PGLYRP2 | ENSG00000161031.12 |
| U91324.1 | ENSG00000229740.1 |
| ABCB4 | ENSG00000005471.15 |
| ITIH2 | ENSG00000151655.17 |
| SLC22A1 | ENSG00000175003.12 |
| DPYS | ENSG00000147647.12 |
| RP11-469A15.2 | ENSG00000230623.2 |
| PON3 | ENSG00000105852.10 |
| AP000355.2 | ENSG00000228923.1 |
| CYP4A11 | ENSG00000187048.12 |
| TTPA | ENSG00000137561.4 |
| CPS1 | ENSG00000021826.14 |
| CA5A | ENSG00000174990.4 |
| RP11-328K4.1 | ENSG00000248740.5 |
| GAMT | ENSG00000130005.11 |
| APCS | ENSG00000132703.3 |
| MTND4P20 | ENSG00000233377.1 |
| GOLT1A | ENSG00000174567.7 |
| RP11-1151B14.3 | ENSG00000267391.2 |
| FGL1 | ENSG00000104760.16 |
| PCK1 | ENSG00000124253.10 |
| CIDEB | ENSG00000136305.11 |
| FTCD | ENSG00000160282.13 |
| AGT | ENSG00000135744.7 |
| IL27 | ENSG00000197272.2 |
| SEC16B | ENSG00000120341.18 |
| HFE2 | ENSG00000168509.17 |
| TMPRSS6 | ENSG00000187045.16 |
| PAQR9 | ENSG00000188582.8 |
| SLC25A15 | ENSG00000102743.14 |
| LINC01093 | ENSG00000249173.5 |
| GLTPD2 | ENSG00000182327.7 |
| PRAP1 | ENSG00000165828.13 |
| REEP6 | ENSG00000115255.10 |
| RNU1-70P | ENSG00000199488.1 |
| RP11-14C10.4 | ENSG00000282793.1 |
| SEPP1 | ENSG00000250722.5 |
| ACSL1 | ENSG00000151726.13 |
| FETUB | ENSG00000090512.11 |
| MBL2 | ENSG00000165471.6 |
| TST | ENSG00000128311.13 |
| BAAT | ENSG00000136881.11 |
| RP11-513G11.3 | ENSG00000238097.1 |
| CFB | ENSG00000243649.8 |
| ADORA2A-AS1 | ENSG00000178803.10 |
| LINC01348 | ENSG00000280587.1 |
| PLA2G12B | ENSG00000138308.5 |
| RDH16 | ENSG00000139547.7 |
| SERPINA6 | ENSG00000170099.5 |
| HORMAD2-AS1 | ENSG00000227117.6 |
| SERPINA11 | ENSG00000186910.3 |
| RGN | ENSG00000130988.12 |
| C3 | ENSG00000125730.16 |
| TMEM56-RWDD3 | ENSG00000271092.5 |
| CYP8B1 | ENSG00000180432.5 |
| UGT2B27P | ENSG00000251685.3 |
| RTP3 | ENSG00000163825.3 |
| CFHR3 | ENSG00000116785.13 |
| HGD | ENSG00000113924.11 |
| AADAC | ENSG00000114771.13 |
| C1S | ENSG00000182326.14 |
| UGT2A3P7 | ENSG00000248886.2 |
| A1BG | ENSG00000121410.11 |
| CFHR4 | ENSG00000134365.12 |
| XXbac-BPG116M5.17 | ENSG00000244255.5 |
| EPHX1 | ENSG00000143819.12 |
| HORMAD2 | ENSG00000176635.17 |
| APOA2 | ENSG00000158874.11 |
| OR10J6P | ENSG00000158731.2 |
| MST1P2 | ENSG00000186301.8 |
| PHYH | ENSG00000107537.13 |
| FUOM | ENSG00000148803.11 |
| RP11-180D21.3 | ENSG00000224943.1 |
| PRODH2 | ENSG00000250799.9 |
| BDH1 | ENSG00000161267.11 |
| F5 | ENSG00000198734.10 |
| FAM99A | ENSG00000205866.3 |
| HSD17B13 | ENSG00000170509.11 |
| HNF4A-AS1 | ENSG00000229005.2 |
| ABCC6 | ENSG00000091262.14 |
| ADH4 | ENSG00000198099.8 |
| SLC25A47 | ENSG00000140107.10 |
| C9 | ENSG00000113600.10 |
| FAM47E-STBD1 | ENSG00000118804.8 |
| KLB | ENSG00000134962.6 |
| AOC4P | ENSG00000260105.6 |
| UROC1 | ENSG00000159650.8 |
| RP11-14C10.4 | ENSG00000277210.3 |
| C4BPA | ENSG00000123838.10 |
| GJB1 | ENSG00000169562.9 |
| RP11-35N6.6 | ENSG00000230328.2 |
| ENPP7 | ENSG00000182156.9 |
| LBP | ENSG00000129988.5 |
| SPP2 | ENSG00000072080.10 |
| HABP2 | ENSG00000148702.14 |
| ERICH5 | ENSG00000177459.10 |
| HPR | ENSG00000261701.6 |
| NR0B2 | ENSG00000131910.4 |
| CYP2D6 | ENSG00000100197.20 |
| RPL7AP34 | ENSG00000213312.3 |
| SLC22A10 | ENSG00000184999.11 |
| PKLR | ENSG00000143627.17 |
| RP11-209K10.2 | ENSG00000259203.1 |
| CDO1 | ENSG00000129596.4 |
| CCL15-CCL14 | ENSG00000275688.4 |
| ABCA6 | ENSG00000154262.12 |
| RP5-968D22.3 | ENSG00000237658.1 |
| HGFAC | ENSG00000109758.8 |
| CCL16 | ENSG00000275152.4 |
| CYP2A6 | ENSG00000255974.6 |
| SMLR1 | ENSG00000256162.2 |
| AHSG | ENSG00000145192.12 |
| FAH | ENSG00000103876.11 |
| SHBG | ENSG00000129214.14 |
| RP11-563D10.1 | ENSG00000227240.1 |
| CYP7A1 | ENSG00000167910.3 |
| HPD | ENSG00000158104.11 |
| MARC2 | ENSG00000117791.15 |
| AKR1C4 | ENSG00000198610.10 |
| GOT1 | ENSG00000120053.10 |
| KRT17P8 | ENSG00000256937.1 |
| PECR | ENSG00000115425.13 |
| IGFBP1 | ENSG00000146678.9 |
| FMO3 | ENSG00000007933.12 |
| CTD-2545H1.2 | ENSG00000262445.3 |
| DDT | ENSG00000099977.13 |
| HMGCS2 | ENSG00000134240.11 |
| AC009166.5 | ENSG00000261238.1 |
| RP11-252E2.2 | ENSG00000261058.1 |
| SLC25A20 | ENSG00000178537.9 |
| DHODH | ENSG00000102967.11 |
| SLC27A2 | ENSG00000140284.10 |
| FAM47E-STBD1 | ENSG00000272414.5 |
| SLC27A5 | ENSG00000083807.9 |
| SLC30A10 | ENSG00000196660.10 |
| SLC22A9 | ENSG00000149742.9 |
| ADH1C | ENSG00000248144.5 |
| APOM | ENSG00000204444.10 |
| FYTTD1P1 | ENSG00000234502.2 |
| ADH6 | ENSG00000172955.17 |
| KDM8 | ENSG00000155666.11 |
| RP11-42O15.3 | ENSG00000271992.1 |
| GALK1 | ENSG00000108479.11 |
| HYAL1 | ENSG00000114378.16 |
| RP13-650J16.1 | ENSG00000264569.1 |
| RP11-573D15.8 | ENSG00000197099.8 |
| TM4SF5 | ENSG00000142484.6 |
| AGMO | ENSG00000187546.13 |
| ACSM5 | ENSG00000183549.10 |
| MPST | ENSG00000128309.16 |
| GGCX | ENSG00000115486.11 |
| F10 | ENSG00000126218.11 |
| TMEM176B | ENSG00000106565.17 |
| LINC01554 | ENSG00000236882.7 |
| ABCB11 | ENSG00000073734.8 |
| FGF21 | ENSG00000105550.8 |
| TTC39C | ENSG00000168234.12 |
| G6PC | ENSG00000131482.9 |
| SPRYD4 | ENSG00000176422.12 |
| THPO | ENSG00000090534.17 |
| INHBE | ENSG00000139269.2 |
| SULT2A1 | ENSG00000105398.3 |
| PLIN5 | ENSG00000214456.8 |
| CTD-2619J13.8 | ENSG00000268230.5 |
| CYP27A1 | ENSG00000135929.8 |
| RP11-484N16.1 | ENSG00000266304.1 |
| RP11-1267H10.3 | ENSG00000251074.1 |
| FMO5 | ENSG00000131781.12 |
| FH | ENSG00000091483.6 |
| SEPHS2 | ENSG00000179918.16 |
| HIST1H2APS2 | ENSG00000242387.1 |
| CYP3A7 | ENSG00000160870.12 |
| CYP3A7-CYP3A51P | ENSG00000282301.1 |
| CES2 | ENSG00000172831.11 |
| RARRES2 | ENSG00000106538.9 |
| OIT3 | ENSG00000138315.12 |
| DGAT2 | ENSG00000062282.14 |
| ACSM2B | ENSG00000066813.14 |
| ECHS1 | ENSG00000127884.4 |
| CYP3A43 | ENSG00000021461.16 |
| AKR1C8P | ENSG00000264006.6 |
| APOE | ENSG00000130203.9 |
| HAGH | ENSG00000063854.12 |
| RP4-710M16.2 | ENSG00000223956.1 |
| UGT1A1 | ENSG00000241635.7 |
| AOX1 | ENSG00000138356.13 |
| F11-AS1 | ENSG00000251165.5 |
| HPN | ENSG00000105707.13 |
| RP11-669E14.4 | ENSG00000262881.1 |
| RP11-468N14.3 | ENSG00000251424.1 |
| TM4SF4 | ENSG00000169903.6 |
| OXER1 | ENSG00000162881.6 |
| CYP3A5 | ENSG00000106258.13 |
| SLC51A | ENSG00000163959.9 |
| ARID3C | ENSG00000205143.2 |
| AL161668.5 | ENSG00000258604.1 |
| ABCC6P1 | ENSG00000256340.8 |
| RP11-799B12.1 | ENSG00000279332.1 |
| ADH1B | ENSG00000196616.12 |
| OSGIN1 | ENSG00000140961.12 |
| ALDH2 | ENSG00000111275.12 |
| APOC4 | ENSG00000267467.3 |
| SLC17A2 | ENSG00000112337.10 |
| PCYT2 | ENSG00000185813.10 |
| ADORA2BP1 | ENSG00000236136.1 |
| CTH | ENSG00000116761.11 |
| AC073842.19 | ENSG00000235077.1 |
| PC | ENSG00000173599.13 |
| ADCY10 | ENSG00000143199.17 |
| RP11-104J23.1 | ENSG00000275944.1 |
| EHHADH | ENSG00000113790.10 |
| TAT-AS1 | ENSG00000260886.1 |
| C4B | ENSG00000224389.8 |
| FAM99B | ENSG00000205865.4 |
| POR | ENSG00000127948.13 |
| GSTA1 | ENSG00000243955.5 |
| GRHPR | ENSG00000137106.17 |
| CTD-2350C19.2 | ENSG00000265254.1 |
| RP11-290F5.1 | ENSG00000249096.6 |
| GPLD1 | ENSG00000112293.14 |
| UGP2 | ENSG00000169764.14 |
| IGSF23 | ENSG00000216588.8 |
| UGT1A4 | ENSG00000244474.5 |
| CES1 | ENSG00000198848.12 |
| C4A | ENSG00000244731.7 |
| SULT1A2 | ENSG00000197165.10 |
| TMEM82 | ENSG00000162460.6 |
| CYP4F3 | ENSG00000186529.14 |
| FBP1 | ENSG00000165140.9 |
| TRIM80P | ENSG00000232724.1 |
| CTB-96E2.2 | ENSG00000273171.1 |
| FABP1 | ENSG00000163586.9 |
| ABCC2 | ENSG00000023839.10 |
| CLDN14 | ENSG00000159261.10 |
| LEPR | ENSG00000116678.18 |
| TMEM110 | ENSG00000213533.11 |
| TTC38 | ENSG00000075234.16 |
| ST6GAL1 | ENSG00000073849.14 |
| GATM | ENSG00000171766.15 |
| LECT2 | ENSG00000145826.8 |
| HS1BP3-IT1 | ENSG00000231948.2 |
| ADI1 | ENSG00000182551.13 |
| CFI | ENSG00000205403.12 |
| AC022816.2 | ENSG00000230647.1 |
| TM6SF2 | ENSG00000213996.12 |
| KARSP2 | ENSG00000230371.1 |
| NAGS | ENSG00000161653.10 |
| CFHR5 | ENSG00000134389.9 |
| PHBP11 | ENSG00000227621.1 |
| ALDH4A1 | ENSG00000159423.16 |
| AC068535.3 | ENSG00000225765.1 |
| ATF5 | ENSG00000169136.8 |
| RP5-888M10.2 | ENSG00000229484.1 |
| DCXR | ENSG00000169738.7 |
| RP11-41O4.1 | ENSG00000266258.1 |
| BTD | ENSG00000169814.12 |
| NADK2 | ENSG00000152620.12 |
| SLC43A1 | ENSG00000149150.8 |
| DECR1 | ENSG00000104325.6 |
| KLF15 | ENSG00000163884.3 |
| XYLB | ENSG00000093217.9 |
| ACY1 | ENSG00000243989.7 |
| SMUG1P1 | ENSG00000267444.1 |
| CEBPA | ENSG00000245848.2 |
| AC008592.4 | ENSG00000251409.1 |
| GLT1D1 | ENSG00000151948.11 |
| RP11-753B14.1 | ENSG00000237494.1 |
| RP11-168L7.1 | ENSG00000258460.1 |
| BHMT2 | ENSG00000132840.9 |
| RP11-434D9.1 | ENSG00000249364.5 |
| BHMT | ENSG00000145692.14 |
| KHK | ENSG00000138030.12 |
| VNN3 | ENSG00000093134.13 |
| HMGCL | ENSG00000117305.14 |
| RP11-101E14.3 | ENSG00000233415.1 |
| ECHDC2 | ENSG00000121310.16 |
| BPHL | ENSG00000137274.12 |
| CYP3AP1 | ENSG00000282277.1 |
| GPAM | ENSG00000119927.13 |
| CYB5A | ENSG00000166347.18 |
| NR5A2 | ENSG00000116833.13 |
| URAHP | ENSG00000222019.7 |
| LPA | ENSG00000198670.11 |
| RP11-740C1.2 | ENSG00000231100.1 |
| LAMA5-AS1 | ENSG00000228812.7 |
| TTC36 | ENSG00000172425.10 |
| PROX1 | ENSG00000117707.15 |
| SCP2 | ENSG00000116171.16 |
| HSD17B2 | ENSG00000086696.10 |
| APOC1P1 | ENSG00000214855.9 |
| CTD-3128G10.7 | ENSG00000276980.1 |
| PRAMEF10 | ENSG00000187545.5 |
| RP11-115J16.1 | ENSG00000254235.5 |
| PQLC1 | ENSG00000122490.18 |
| GSTA2 | ENSG00000244067.2 |
| PNPLA3 | ENSG00000100344.10 |
| GNMT | ENSG00000124713.5 |
| RP11-872D17.8 | ENSG00000254979.5 |
| NR1H4 | ENSG00000012504.13 |
| TKFC | ENSG00000149476.14 |
| ACOX2 | ENSG00000168306.12 |
| ACMSD | ENSG00000153086.13 |
| ECI2 | ENSG00000198721.12 |
| ACAA1 | ENSG00000060971.17 |
| RP4-763G1.2 | ENSG00000235200.1 |
| CHST13 | ENSG00000180767.9 |
| RP11-96D1.6 | ENSG00000261469.1 |
| NBPF13P | ENSG00000227242.3 |
| RP4-782L23.1 | ENSG00000231816.1 |
| CD14 | ENSG00000170458.13 |
| SLC16A13 | ENSG00000174327.6 |
| PPP2R1B | ENSG00000137713.15 |
| HSD11B1 | ENSG00000117594.9 |
| RP4-798P15.3 | ENSG00000254154.8 |
| LDHD | ENSG00000166816.13 |
| IGHV1OR15-4 | ENSG00000270356.1 |
| RP11-361C13.1 | ENSG00000280655.1 |
| CCL15 | ENSG00000275718.1 |
| RP11-555M1.3 | ENSG00000241770.1 |
| CTD-2619J13.5 | ENSG00000279611.1 |
| RP11-15I11.3 | ENSG00000226251.5 |
| ECHDC3 | ENSG00000134463.14 |
| LPAL2 | ENSG00000213071.10 |
| CTD-2619J13.27 | ENSG00000273901.1 |
| MOGAT3 | ENSG00000106384.10 |
| TMEM176A | ENSG00000002933.7 |
| OAF | ENSG00000184232.8 |
| RP11-219C24.6 | ENSG00000237700.1 |
| TCP10L | ENSG00000242220.6 |
| GLYATL1 | ENSG00000166840.13 |
| RP11-700H6.4 | ENSG00000262006.1 |
| ETNK2 | ENSG00000143845.14 |
| RP11-38L15.8 | ENSG00000272430.1 |
| LINC01370 | ENSG00000237767.1 |
| ETFDH | ENSG00000171503.11 |
| RP4-583P15.14 | ENSG00000273047.1 |
| UPP2 | ENSG00000007001.12 |
| RP11-485O10.2 | ENSG00000259670.1 |
| ETFA | ENSG00000140374.15 |
| RNASE4 | ENSG00000258818.3 |
| C19orf80 | ENSG00000130173.13 |
| SLCO2B1 | ENSG00000137491.14 |
| DKFZp779M0652 | ENSG00000205106.4 |
| HAO2 | ENSG00000116882.14 |
| APOL5 | ENSG00000128313.2 |
